# Impact of blade direction on postoperative femoral head varus in PFNA fixed patients: a clinical review and biomechanical research

**DOI:** 10.3389/fbioe.2024.1381201

**Published:** 2024-07-12

**Authors:** Yue Xu, Xiaoyu Zhang, Jingchi Li, Yiming Miao, Pu Ying, Cong Chen, Wenqiang Xu, Qiang Wang

**Affiliations:** ^1^ Department of Orthopaedics, Changshu Hospital Affiliated to Nanjing University of Chinese Medicine, Changshu, Jiangsu, China; ^2^ Department of Orthopedics, Affiliated Hospital of Integrated Traditional Chinese and Western Medicine, Nanjing University of Chinese Medicine, Nanjing, Jiangsu, China; ^3^ Department of Orthopedics, Luzhou Key Laboratory of Orthopedic Disorders, The Affiliated Traditional Chinese Medicine Hospital, Southwest Medical University, Luzhou, Sichuan, China

**Keywords:** proximal femoral nail anti-rotation, intertrochanteric fracture, femoral head varus, clinical review, biomechanical simulations

## Abstract

Intertrochanteric femur fracture is a common type of osteoporotic fracture in elderly patients, and postoperative femoral head varus following proximal femoral nail anti-rotation (PFNA) fixation is a crucial factor contributing to the deterioration of clinical outcomes. The cross-angle between the implant and bone might influence fixation stability. Although there is a wide range of adjustment in the direction of anti-rotation blades within the femoral neck, the impact of this direct variation on the risk of femoral head varus and its biomechanical mechanisms remain unexplored. In this study, we conducted a retrospective analysis of clinical data from 69 patients with PFNA fixation in our institution. We judge the direction of blade on the femoral neck in on the immediate postoperative lateral X-rays or intraoperative C-arm fluoroscopy, investigating its influence on the early postoperative risk of femoral head varus. *p* < 0.05 indicates significant results in both correlation and regression analyses. Simultaneously, a three-dimensional finite element model was constructed based on the Syn-Bone standard proximal femur outline, exploring the biomechanical mechanisms of the femoral neck-anti-rotation blade direction variation on the risk of this complication. The results indicated that ventral direction insertion of the anti-rotation blade is an independent risk factor for increased femoral head varus. Complementary biomechanical studies further confirmed that ventral angulation leads to loss of fixation stability and a decrease in fixation failure strength. Therefore, based on this study, it is recommended to avoid ventral directional insertion of the anti-rotation blade in PFNA operation or to adjust it in order to reduce the risk of femoral head varus biomechanically, especially in unstable fractures. This adjustment will help enhance clinical outcomes for patients.

## Background

Intertrochanteric fracture is a typical osteoporotic fracture in the elderly, and its incidence risk is gradually increasing with the aging population in China (Johnell and Kanis, 2005; Lane, 2006). Due to its high mortality rate, it is referred to as the “last fracture in life,” leading to a significant economic and social burden (Weil et al., 2012; Randelli et al., 2023). Internal fixation surgery is an effective means of treating intertrochanteric fractures of the femur (Haidukewych, 2010; Ricci, 2023). Over the past few decades, various types of internal fixation have been applied in the treatment of intertrochanteric fractures, achieving certain therapeutic effects. Among them, the Proximal Femoral Nail Antirotation (PFNA), with its simple operating procedure and good fixation stability, has become the most widely used internal fixation method in the surgical treatment of this condition (Li et al., 2019; Nie et al., 2022). However, postoperative varus collapse of the femoral head and cutout of the implant remain significant factors contributing to deteriorated clinical outcomes for PFNA fixed patients.

Studies indicate that the loss of fixation stability and stress concentration at the bone-screw interface are important factors leading to postoperative femoral head varus and fixation failure (Nikoloski et al., 2013; Nie et al., 2022). The potential risk factors can be categorized into two classes: patients’ demographic factors and surgical related factors. Regarding patient-related factors, the progression of osteoporosis and the presence of unstable fracture types are identified as causes of postoperative femoral head varus (Blake and Fogelman, 2007; Armas and Recker, 2012). As for surgical operation factors, nail length, tip-apex distance (TAD), and the relative position of the anti-rotation blade in the neutral position have also been proven to contribute to the increased risk of the complication (Rubio-Avila et al., 2013; Coviello et al., 2024).

The orientation of the internal fixation device in relation to bony structures can have an impact on the stability of fixation by altering the postoperative biomechanical environment, which in turn may affect immediate postoperative stability (Amirouche et al., 2016; Fletcher et al., 2019). Theoretically, the anti-rotation blade should be aligned parallel to the axis of the femoral neck. However, the direction of blade insertion is highly adjustable. Despite this, there is a lack of published studies identifying the biomechanical significance of changes in blade insertion direction on fixation stability. Based on above theoretical and practical foundations, we hypothesize that changes in blade insertion direction can affect femoral head varus biomechanically. This study aims to comprehensively investigate this issue through clinical and biomechanical research, with the goal of providing insights for optimizing PFNA technique and improving patient outcomes following fixation. To our knowledge, this is the first study to address this topic.

## Material and methods

### Clinical data review

#### Collection of patient medical records

This study was conducted with the approval of our hospital’s ethics committee. As this is a retrospective analysis, patient informed consent was waived. Clinical data of patients who underwent PFNA fixation surgery for intertrochanteric fractures from January 2019 to January 2021, at our hospital were retrospectively collected for analysis. Using the hospital’s medical records system, baseline information of patients (gender, age, BMI) was retrospectively recorded. Dual-energy X-ray scan-derived T-values were documented to assess patient bone density (BMD). Exclusion criteria for patients were as follows: 1. Patients with subtrochanteric fractures (i.e., AO-3.1 A3-type fractures); 2. Patients who died during the follow-up period; 3. Lost to follow-up patients; 4. Patients with pathological fractures due to tumors or rheumatoid inflammation; 5. Patients treated conservatively; 6. Patients who remained bedridden for an extended period after surgery due to other underlying diseases, with no weight-bearing on the lower limbs. Clinical data from 69 patients (28 Male, 41 Female), with average age = 73.87 ± 14.58 years were collected in this study. BMD of these patients ranged from −1.1 to −4,1. The incidence rate of osteoporosis (i.e., T ≤ 2.5) was 63.77% (44/69).

#### Measurement of radiological indicators

**FIGURE 1:**
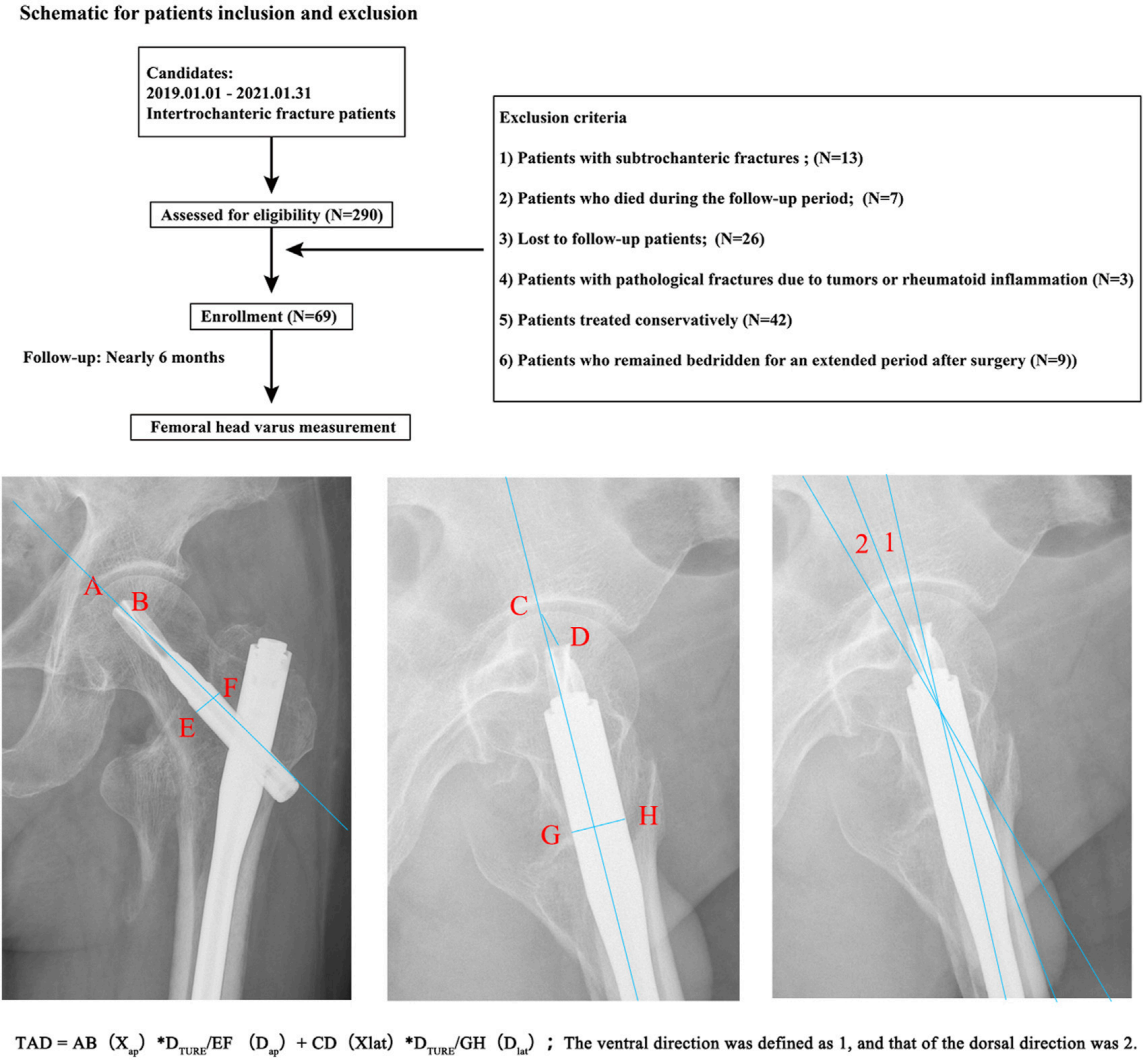
Patient inclusion and exclusion protocol, and the measurement of TAD, femoral head varus, and blade directions. Compared to the quantitative definition of blade insertion angle, the confounidng effect of imaging angles on the dichotomous blade insertion directions definition was limited.

All radiological measurements were independently conducted by an orthopedic physician with extensive experience in interpreting orthopedic imaging. The anti-rotation blade tip-apex distance (TAD) was measured on immediate postoperative X-ray radiographs (Nikoloski et al., 2013; Rubio-Avila et al., 2013). The neck-shaft angle of the affected limb was measured on anteroposterior radiographs at both immediate postoperative and 6-month follow-up visits, with the difference in neck-shaft angles calculated as the amount of femoral head varus (Nikoloski et al., 2013; Nie et al., 2022). The ventral and dorsal directional insertion of anti-rotation blade has been judged on immediate postoperative (or intraoperative C-arm fluoroscopy) lateral radiographs (Born et al., 2011; Chang et al., 2020). The ventral direction was defined as 1, and that of the dorsal direction was 2, separately (Figure 1).

#### Statistical Analysis

Statistical Analysis have been performed in the SPSS 26.0 in this study. One week after completing the radiological data measurements, a randomly selected imaging specialist with extensive experience in orthopedic imaging and the aforementioned orthopedic physician re-evaluated the imaging data for 20 patients to assess inter-rater reliability (Li J. et al., 2022; Li J. C. et al., 2022). For binary variables (fracture stability), Kappa coefficients were utilized to assess their consistency (Weishaupt et al., 1999; Pfirrmann et al., 2001). For continuous variables, Intraclass Correlation Coefficients (ICC) were used to measure their consistency. Normality tests were conducted for all continuous variables (Li J. et al., 2022; Li et al., 2023). Descriptive statistics were presented in the form of mean ± standard deviation for variables conforming to a normal distribution. For non-normally distributed continuous variables, descriptive statistics were presented using the four-category (25%, 50%, 75%) method.

For binary variables (gender, fracture stability), proportions were used for description. In correlation analysis, each variable was correlated with the amount of femoral head varus collapse. PEARSON correlation coefficients were used for normally distributed continuous variables, while SPEARMAN correlation coefficients were used for binary data and non-normally distributed variables (Hsieh et al., 2019; Chang et al., 2021). A significance level of *p* < 0.05 indicated a significant correlation between two variables. Linear regression analysis was employed to explore potential independent risk factors for femoral head varus collapse. In univariate regression, each variable was individually included, and indicators with *p* < 0.1 were incorporated into multivariate regression. In multivariate regression, variables with *p* < 0.05 were considered independent risk factors leading to femoral head varus (Li et al., 2023; Xi et al., 2023).

### Mechanical analysis

#### Reconstruction of intertrochanteric fracture model

The SYN-BONE femoral outline model was selected for model reconstruction. The SYN-BONE model was scanned using a 128-slice spiral CT with a scan thickness set at 0.55 mm. After scanning, the model’s outer contour was constructed in 3D-CAD software using a forward drafting method to eliminate interference from irregular surfaces on the analysis results. For the modeling of the intertrochanteric fracture model, following the methodology of similar studies, an A2.3-type unstable intertrochanteric fracture model was constructed. The specific modeling method involved creating the fracture by intersecting three fracture lines (Chen et al., 2013; Mao et al., 2023). The first fracture line was positioned 10 mm below the greater trochanter, forming a 20 angle with the long axis of the femoral shaft. The second fracture line was set tangent to the upper edge of the lesser trochanter, and the third fracture line connected the intersection of the first and second lines with the vertex of the greater trochanter (Li et al., 2019; Nie et al., 2022). The bone within the cut range was removed to complete the reconstruction of the fracture model (Liang et al., 2018; Hamidi et al., 2021).

#### Construction of PFNA fixation model

**FIGURE 2:**
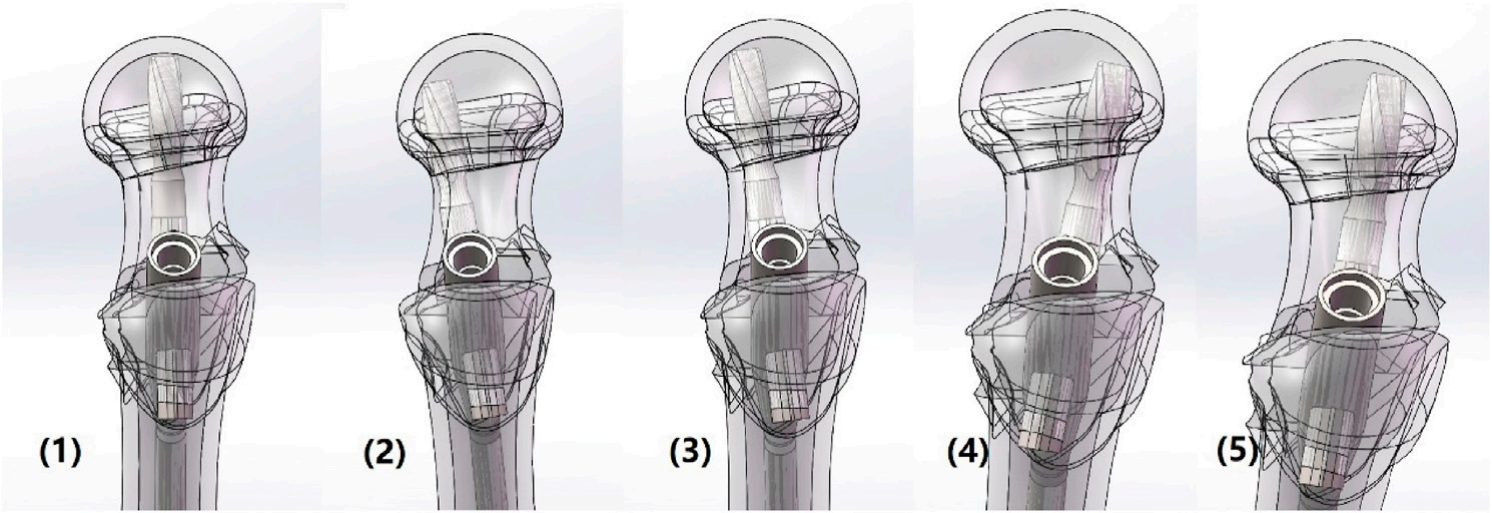
Model construction strategies of PFNA fixation with different ventral and dorsal blade insertion.

In the PFNA fixation model, the entry point of the main nail was positioned at the center of the femoral shaft. The anti-rotation blade was set parallel to the long axis of the femoral neck in both the sagittal and coronal planes, and it was positioned at the midline of the long axis of the femoral neck (Lewis et al., 2021; Luque Pérez et al., 2022). This model was designated as the original control group model, and all subsequent models were adjusted based on this original model (Model. 1). To construct different femoral neck-anti-rotation blade intersection angle models, adjustments were made to the anti-rotation blade angle as follows: Model. 2: Anti-rotation blade counterclockwise rotation, close to the posterior cortical bone; Model. 3: Anti-rotation blade counterclockwise rotation, blade angle set to the midpoint between Model 1 and the original control group model; Model. 4: Anti-rotation blade clockwise rotation, close to the anterior cortical bone; Model. 5: Anti-rotation blade clockwise rotation, blade angle set to the midpoint between Model 4 and the original control group model. Schematic for the model construction strategy has been presented in the Figure 2.

#### Boundary and loading conditions

Numerical simulations for this study were carried out using “Ansys Workbench 2020 R2 Academic”. The lower surface of the proximal femur model was completely constrained in all degrees of freedom, while the load was applied to the upper surface of the femoral head. The loading direction was 10° abduction in the coronal plane and 9° extension in the sagittal plane (Li et al., 2019; Nie et al., 2022). Tetrahedral meshes of varying sizes were comprehensively applied to complete the meshing. Mesh refinement was performed in regions of high stress and large deformation to improve mesh convergence and prevent analysis errors caused by mesh distortion.

The friction coefficient at the bone-implant interface was defined as 0.2, with a firm contact defined between the implants (Li J. C. et al., 2022; Yang et al., 2024). The load was incrementally increased from 0N, with steps of 300N, up to 2100N. Throughout this process, the peak displacement of the femoral head was recorded (Li et al., 2019; Nie et al., 2022). The displacement at the final loading step and the load when the femoral head displacement reached 10 mm were collected and defined as failure load (Li et al., 2019; Nie et al., 2022). According to similar studies, these two parameters can effectively assess the fixation stability of PFNA operation and predict potential risk of fixation failure (Li et al., 2019; Nie et al., 2022).

## Results

### Clinical review and the judgement of independent risk factors for femoral head varus in PFNA fixed patients

**FIGURE 3:**
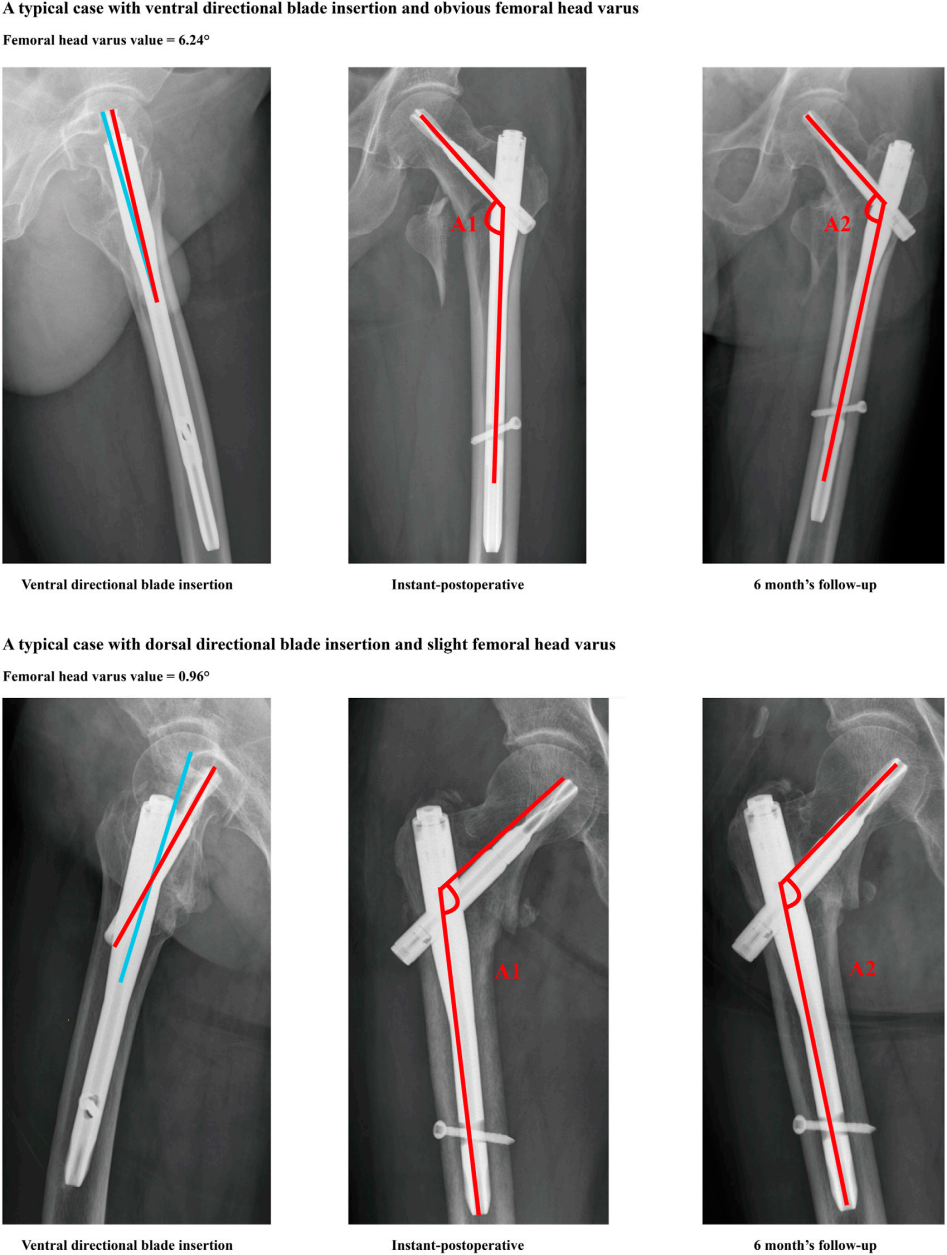
Typical cases for blade ventral directional insertion and severe femoral head varus, and blade dorsal directional insertion and slight femoral head varus. Based on the regression analysis, compared to the dorsal directional blade insertion, ventral direction blade insertion can trigger higher incidence of femoral head varus progression.

Excellent intra- and inter-observer measurement of imaging-based parameters was assessed through the computation of ICC and Kappa values (Table 1). The correlation analysis revealed a significant correlation between increased TAD (*p* = 0.006), ventral directional insertion of the anti-rotation blade (*p* = 0.000), decreased T-values (*p* = 0.036), and increased femoral head varus collapse. Furthermore, multivariate linear regression analysis confirmed that poor BMD (*p* = 0.046) and ventral directional blade insertion (*p* = 0.000) were independent risk factors for an increased risk of femoral head varus collapse. Other factors did not show a significant correlation with an increased femoral head varus collapse and were not identified as independent risk factors (Figure 3; Tables 2, 3).

**TABLE 1:** ICC and Kappa values of inter- and intraobserver reliability when measuring imaging based parameters.

	Interobserver	Intraobserver
Blade directions	0.904	0.815
Femoral head varus values	0.872	0.883
TAD values	0.864	0.855

**TABLE 2:** Correlation coefficients between femoral head varus and variates.

Femoral head varus	Correlation coefficients	*p*-value
Blade positions (Ventral direction: 1, dorsal direction: 2)	−0.571	0.000**
TAD	0.327	0.006**
Age	−0.172	0.158
Sex (Male: 1, Female: 2)	−0.165	0.177
BMI	0.182	0.134
BMD	−0.253	0.036*

*Statistical significance (*p* < 0.05).

**Statistical significance (*p* < 0.01).

**TABLE 3:** Linear regression analysis of severe femoral head varus.

	t	95% CI		*p*-value
Uni-variable analyses				
Blade positions (Ventral direction: 1, dorsal direction: 2)	−4.623	−3.183	−1.263	0.000^#^
TAD	2.834	0.038	0.22	0.006^#^
Age	−1.428	−0.064	0.011	0.158
Sex (Male: 1, Female: 2)<	−1.662	0.101	−2.011	0.184
BMI	1.515	−0.052	0.38	0.134
BMD	−2.138	−1.274	−0.044	0.036^#^
Multi-variable analyses				
Blade positions	−3.756	−2.856	−0.873	0.000**
TAD	1.62	−0.016	0.155	0.11
BMD	−2.039	−1.085	−0.011	0.046*

^#^Variables that achieved a significance level of *p* < 0.1 in the univariate analysis.

*Statistical significance (*p* < 0.05).

**Statistical significance (*p* < 0.01).

### Fixation strength computation

**FIGURE 4:**
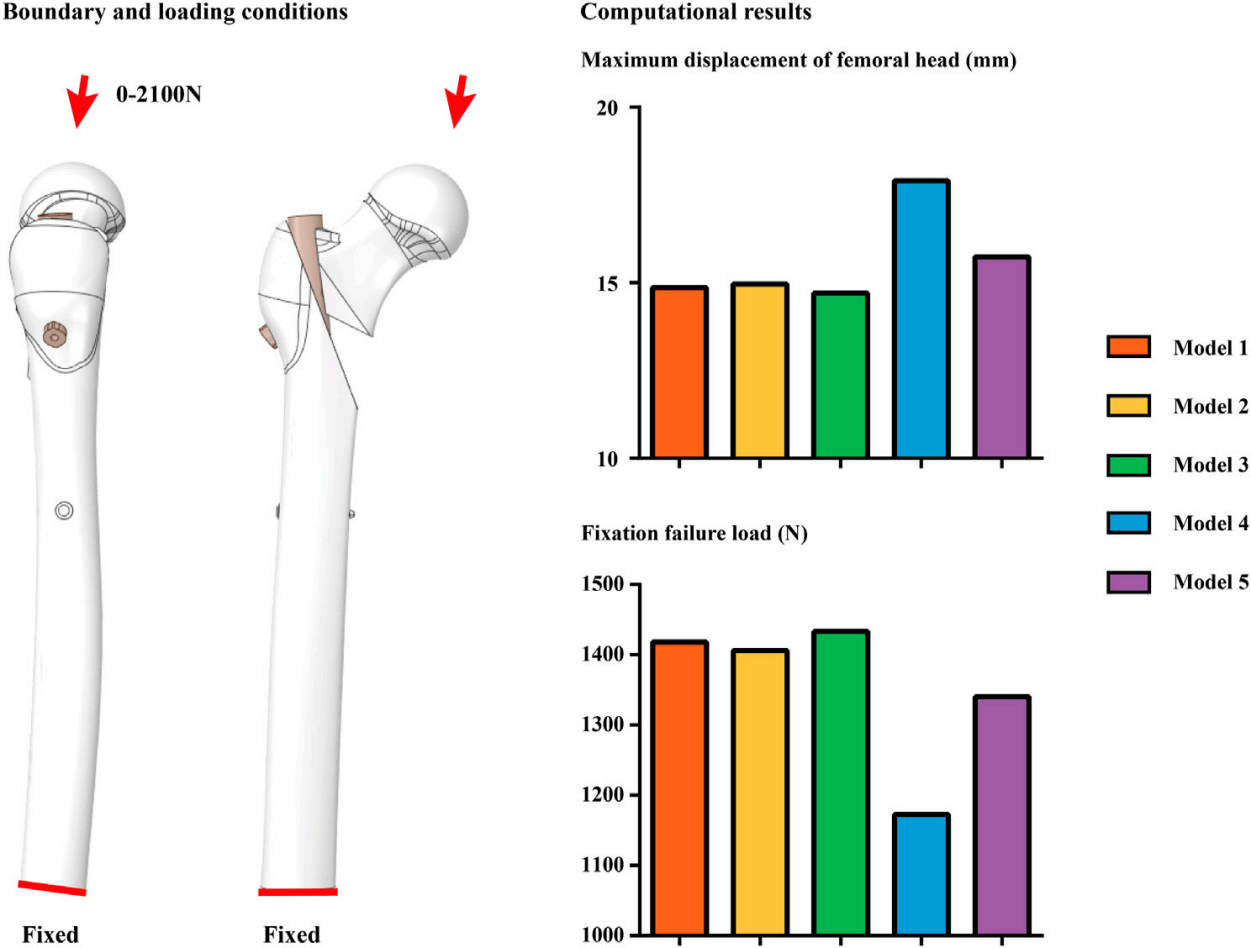
Boundary and loading conditions for models computation, and computational results in different models. Compared to the dorsal directional blade insertion, ventral blade insertion can trigger poor fixation stability. This can explain the clinically observed phenomenon.

The biomechanical study revealed that the maximum displacement of the femoral head in Models 4 and 5 was significantly higher than in the other three models, and the failure load was obviously lower than in the other three groups. Compared to the model 1 (PFNA fixed model whose anti-rotation blade was parallel to the femoral neck in the lateral radiography), the maximum femoral head displacement value of the model whose anti-rotation blade clockwise rotation, close to the anterior cortical bone; increased by more than 20%, and that of the failure load decreased by 17.32%. In the model 5 (the model whose blade clockwise rotation, blade angle set to the midpoint between Model 4 and the model 1), the femoral head displacement value increased by nearly 5%, and that of the failure load also decreased by nearly 5%. Moreover, differences in computed parameters between the model 1 and models whose blade anticlockwise rotation (i.e., model 2 and model 3) was nearly 1%. Therefore, consistent with the clinical findings, the biomechanical analysis demonstrated that the counterclockwise rotation of the anti-rotation blade towards the ventral side increased the potential biomechanical risks of femoral head fixation failure and varus collapse (Figure 4; Table 4).

**TABLE 4:** Computational result of the numerical simulation.

Femoral head varus	Maximum displacement of femoral head (mm)	Fixation failure load (N)
Model. 1	14.866	1,418.2
Model. 2	14.966	1,406.12
Model. 3	14.708	1,433.39
Model. 4	17.903	1,172.65
Model. 5	15.732	1,340.11

## Discussion

PFNA fixation is one of the most widely used procedures for treating intertrochanteric fractures of the femur and has shown significant clinical efficacy in the majority of patients. However, postoperative femoral head varus collapse remains a crucial factor leading to worsened clinical outcomes (Frei et al., 2012; Nikoloski et al., 2013). Given that the loss of fixation stability is a biomechanical mechanism behind femoral head varus collapse, and variations in the direction between the implant and bone may contribute to changes in stability (Demir and Camuşcuz, 2012; Alkaly and Bader, 2016). We proposed and validated the hypothesis that “changes in the blade insertion direction may affect the potential risk of femoral head varus collapse after fixation”.

In this study, during the clinical review, we observed that the counterclockwise rotation of the anti-rotation blade towards the ventral side posed a potential risk factor for increasing the likelihood of femoral head varus collapse. In our complementary biomechanical study, we noted poorer fixation stability in the PFNA model with ventrally rotated anti-rotation blades. The consistent findings between the clinical and biomechanical studies confirm that intraoperative maneuvers leading to a loss of fixation stability may elevate the risk of femoral head varus collapse. While there is a potential limitation in terms of quantitative validation of numerical models in this study, it is important to note that our comprehensive biomechanical research, which includes both clinical review and biomechanical simulations, mutually supports and verifies our results. As such, while there may be a lack of precise quantitative results as part of our qualitative analysis, it does not diminish the reliability or validity of the conclusions drawn from this study.

Meanwhile, from the biomechanical perspective, we opted for a modeling strategy focused on unstable intertrochanteric femur fractures. This choice was made because analyzing stability in a fracture type prone to femoral head varus and fixation failure holds greater clinical significance (Haidukewych, 2010; Chang et al., 2020; Ricci, 2023). Consistent with previous research, the clinical section of our study also affirmed that unstable fractures independently contribute to the exacerbation of femoral head varus (Hsueh et al., 2010; Knobe et al., 2013; Rinehart et al., 2021). However, this experimental design is not without its limitations. Specifically, based on the conclusions of our study, surgeons should strive to minimize ventral angulation when inserting anti-rotation blades. In the case of unstable intertrochanteric femur fractures, timely adjustments of ventrally angled anti-rotation blades during surgery are crucial to reduce the risk of femoral head varus and enhance stability.

Although the larger TAD value was not proved to be an independent risk factor for larger femoral head varus in the multi-variable regression analysis, significantly correlation can still be observed between these parameters. Given that the significance of larger TAD on the deterioration of fixation stability have been repeatedly validated by the same type studies, we believe the critical positive result of TAD in the multi-variable regression analysis was root in the limited sample size, and which should be further validated in our future studies. Moreover, while this study has arrived at relatively reliable conclusions through consistent comparisons between clinical research and biomechanical experiments, we acknowledge certain methodological shortcomings, or at least, areas for improvement. Firstly, the angle formed between the femoral neck and the anti-rotation blade is based on intraoperative fluoroscopy or immediate postoperative lateral X-ray examinations. Although this approach offers a convenient and precise measurement of the relative angle between the blade and the femoral neck, the shooting angle still potentially affects the measurement results. In future work, we intend to enhance accuracy by incorporating postoperative immediate CT scans to further refine angle measurements (Choi et al., 2016; Gausden et al., 2017).

Besides, as a case comparative study, patient series in the current study was enrolled from the retrospective review. And given that CT scan was not routinely performed in these patients, we can not get enough patient samples with CT imaging data. In contrast, DXA was routinely examined in these patients. Therefore, T-score was selected to judge patients BMD in this study. This may lead to the analysis results being influenced by pathological bone formation anomalies, causing distortions in the analysis. Recent research has attempted to precisely evaluate proximal femoral bone density through hounsfield unit (HU) measurements of the contralateral femoral neck in preoperative dual hip joint CT scans. This measurement approach might assist in eliminating interference caused by two-dimensional images and pathological bone formation in DXA examinations, offering a more accurate assessment of changes in patient bone density. Therefore, appropriately increasing CT scans during the patient follow-up period may help mitigate the shortcomings of two-dimensional plain film examinations, further substantiating the conclusions drawn in this study.

Theoretically, a quantitative definition of blade insertion angle can more accurately reflect the current main research topic. However, in this study, only dichotomous blade insertion directions (i.e., ventral and dorsal blade insertion directions) have been assessed for the following reasons. The measurement of the angle between the femoral neck and the anti-rotation blade may be influenced by changes in imaging angles. Therefore, to eliminate or at least reduce the confounding effect caused by this factor, we utilized a dichotomous definition of blade direction in the imaging data measurement instead of a quantitative one. The repeatability of the measurement results has been re-validated by computing intra- and inter-observer Kappa values. Thus, although this limitation still exists, any resulting confounding effects can be effectively overcome. Furthermore, precise control over angle during the blade insertion process is difficult to achieve compared to selecting ventral or dorsal blade trajectories. We believe that this study provides a feasible operational strategy for PFNA fixation procedures. Therefore, this limitation does not diminish the clinical significance of our current study. In addition, we plan to perform CT scans in our future perspective studies to re-validate our current research conclusions.

Finally, only the unstable fracture type was selected for this study. We believe that the current numerical model construction strategy can help to avoid potential risks of obtaining false negative results caused by stable fracture types. The necessity of making such adjustments for patients with stable intertrochanteric femur fractures remains undocumented in current research. Therefore, subsequent studies should include a separate analysis of patients with stable fractures and construct relevant biomechanical models to elucidate this issue. This will ultimately help to avoid unnecessary prolongation of surgical experiments, increased fluoroscopy sessions, and higher blood loss when adjusting the blade insertion direction.

## Conclusion

Through a comprehensive research consisting of clinical review and numerical mechanical simulations, this study has demonstrated that ventral directional blade insertion can exacerbate femoral head varus in PFNA fixed patients by deteriorating the local biomechanical environment. As a result, the conclusion of this study suggests that ventral direction of blade insertion should be avoided in PFNA fixation, particularly in unstable fractures, to improve clinical outcomes for patients. Despite the limitations mentioned above, this study still offers an innovative perspective for PFNA optimization. Furthermore, our future studies will continue to investigate surgical optimization by conducting comprehensive clinical reviews and biomechanical numerical simulations in patient series with more complete imaging data.

## Data Availability

The raw data supporting the conclusions of this article will be made available by the authors, without undue reservation.

## References

[B1] Alkaly R. N. Bader D. L. (2016). The effect of transpedicular screw design on its performance in vertebral bone under tensile loads: a parametric study. Clin. Spine Surg. 29 (10), 433–440. 10.1097/bsd.0b013e3182a03c70 27879505

[B2] Amirouche F. Solitro G. F. Magnan B. P. (2016). Stability and spine pedicle screws fixation strength-A comparative study of bone density and insertion angle. Spine Deform. 4 (4), 261–267. 10.1016/j.jspd.2015.12.008 27927514

[B3] Armas L. A. Recker R. R. (2012). Pathophysiology of osteoporosis: new mechanistic insights. Endocrinol. Metab. Clin. North Am. 41 (3), 475–486. 10.1016/j.ecl.2012.04.006 22877425

[B4] Blake G. M. Fogelman I. (2007). The role of DXA bone density scans in the diagnosis and treatment of osteoporosis. Postgrad. Med. J. 83 (982), 509–517. 10.1136/pgmj.2007.057505 17675543 PMC2600106

[B5] Born C. T. Karich B. Bauer C. Von Oldenburg G. Augat P. (2011). Hip screw migration testing: first results for hip screws and helical blades utilizing a new oscillating test method. J. Orthop. Res. 29 (5), 760–766. 10.1002/jor.21236 20830738

[B6] Chang H. K. Ku J. Ku J. Kuo Y. H. Chang C. C. Wu C. L. (2021). Correlation of bone density to screw loosening in dynamic stabilization: an analysis of 176 patients. Sci. Rep. 11 (1), 17519. 10.1038/s41598-021-95232-y 34471158 PMC8410763

[B7] Chang S. M. Hou Z. Y. Hu S. J. Du S. C. (2020). Intertrochanteric femur fracture treatment in asia: what we know and what the world can learn. Orthop. Clin. North Am. 51 (2), 189–205. 10.1016/j.ocl.2019.11.011 32138857

[B8] Chen D. W. Lin C. L. Hu C. C. Tsai M. F. Lee M. S. (2013). Biomechanical consideration of total hip arthroplasty following failed fixation of femoral intertrochanteric fractures - a finite element analysis. Med. Eng. Phys. 35 (5), 569–575. 10.1016/j.medengphy.2012.06.023 22824727

[B9] Choi M. K. Kim S. M. Lim J. K. (2016). Diagnostic efficacy of Hounsfield units in spine CT for the assessment of real bone mineral density of degenerative spine: correlation study between T-scores determined by DEXA scan and Hounsfield units from CT. Acta Neurochir. (Wien) 158 (7), 1421–1427. 10.1007/s00701-016-2821-5 27177734

[B10] Coviello M. Abate A. Vicenti G. Ippolito F. Nappi V. Abbaticchio A. M. (2024). Comparison of cutout risk factors between single- and doublescrew proximal nails in intertrochanteric femur fractures - a multicentric study. Med. Glas. (Zenica) 21 (1), 208–213. 10.17392/1683-23 38341752

[B11] Demir T. Camuşcuz N. (2012). Design and performance of spinal fixation pedicle screw system. Proc. Inst. Mech. Eng. H. 226 (1), 33–40. 10.1177/0954411911427351 22888582

[B12] Fletcher J. W. A. Windolf M. Richards R. G. Gueorguiev B. Varga P. (2019). Screw configuration in proximal humerus plating has a significant impact on fixation failure risk predicted by finite element models. J. Shoulder Elb. Surg. 28 (9), 1816–1823. 10.1016/j.jse.2019.02.013 31036421

[B13] Frei H. C. Hotz T. Cadosch D. Rudin M. Käch K. (2012). Central head perforation, or "cut through," caused by the helical blade of the proximal femoral nail antirotation. J. Orthop. Trauma 26 (8), e102–e107. 10.1097/bot.0b013e31822c53c1 22357090

[B14] Gausden E. B. Nwachukwu B. U. Schreiber J. J. Lorich D. G. Lane J. M. (2017). Opportunistic use of CT imaging for osteoporosis screening and bone density assessment: a qualitative systematic review. J. Bone Jt. Surg. Am. 99 (18), 1580–1590. 10.2106/jbjs.16.00749 28926388

[B15] Haidukewych G. J. (2010). Intertrochanteric fractures: ten tips to improve results. Instr. Course Lect. 59, 503–509. 20415401

[B16] Hamidi S. Khosravifard A. Hematiyan M. R. Dehghani J. (2021). A comparative mechanical study of two types of femur bone implant using the finite element method. Int. J. Numer. Method Biomed. Eng. 37 (6), e3459. 10.1002/cnm.3459 33773056

[B17] Hsieh M. K. Liu M. Y. Chen J. K. Tsai T. T. Lai P. L. Niu C. C. (2019). Biomechanical study of the fixation stability of broken pedicle screws and subsequent strategies. PLoS One 14 (6), e0219189. 10.1371/journal.pone.0219189 31251780 PMC6599116

[B18] Hsueh K. K. Fang C. K. Chen C. M. Su Y. P. Wu H. F. Chiu F. Y. (2010). Risk factors in cutout of sliding hip screw in intertrochanteric fractures: an evaluation of 937 patients. Int. Orthop. 34 (8), 1273–1276. 10.1007/s00264-009-0866-2 19784649 PMC2989068

[B19] Johnell O. Kanis J. (2005). Epidemiology of osteoporotic fractures. Osteoporos. Int. 16 (Suppl. 2), S3–S7. 10.1007/s00198-004-1702-6 15365697

[B20] Knobe M. Gradl G. Ladenburger A. Tarkin I. S. Pape H. C. (2013). Unstable intertrochanteric femur fractures: is there a consensus on definition and treatment in Germany? Clin. Orthop. Relat. Res. 471 (9), 2831–2840. 10.1007/s11999-013-2834-9 23389806 PMC3734428

[B21] Lane N. E. (2006). Epidemiology, etiology, and diagnosis of osteoporosis. Am. J. Obstet. Gynecol. 194 (2 Suppl. l), S3–S11. 10.1016/j.ajog.2005.08.047 16448873

[B22] Lewis G. S. Mischler D. Wee H. Reid J. S. Varga P. (2021). Finite element analysis of fracture fixation. Curr. Osteoporos. Rep. 19 (4), 403–416. 10.1007/s11914-021-00690-y 34185266 PMC8422380

[B23] Li J. Han L. Zhang H. Zhao Z. Su X. Zhou J. (2019). Medial sustainable nail versus proximal femoral nail antirotation in treating AO/OTA 31-A2.3 fractures: finite element analysis and biomechanical evaluation. Injury 50 (3), 648–656. 10.1016/j.injury.2019.02.008 30827705

[B24] Li J. Xie Y. Sun S. Xue C. Xu W. Xu C. (2023). Regional differences in bone mineral density biomechanically induce a higher risk of adjacent vertebral fracture after percutaneous vertebroplasty: a case-comparative study. Int. J. Surg. 101 (3), 352–363. 10.1097/js9.0000000000000273 PMC10389488 36912508

[B25] Li J. Zhang Z. Xie T. Song Z. Song Y. Zeng J. (2022). The preoperative Hounsfield unit value at the position of the future screw insertion is a better predictor of screw loosening than other methods. Eur. Radiol. 33, 1526–1536. 10.1007/s00330-022-09157-9 36241918 PMC9935714

[B26] Li J. C. Xie T. H. Zhang Z. Song Z. T. Song Y. M. Zeng J. C. (2022). The mismatch between bony endplates and grafted bone increases screw loosening risk for OLIF patients with ALSR fixation biomechanically. Front. Bioeng. Biotechnol. 10, 862951. 10.3389/fbioe.2022.862951 35464717 PMC9023805

[B27] Liang C. Peng R. Jiang N. Xie G. Wang L. Yu B. (2018). Intertrochanteric fracture: association between the coronal position of the lag screw and stress distribution. Asian J. Surg. 41 (3), 241–249. 10.1016/j.asjsur.2017.02.003 28366494

[B28] Luque Pérez R. Checa Betegón P. Galán-Olleros M. Arvinius C. Valle-Cruz J. Marco F. (2022). Nailing unstable pertrochanteric fractures: does size matters? Arch. Orthop. Trauma Surg. 142 (1), 145–155. 10.1007/s00402-020-03668-0 33146752

[B29] Mao W. Chang S. M. Zhang Y. Q. Li Y. Du S. C. Hu S. J. (2023). Positive medial cortical support versus anatomical reduction for trochanteric hip fractures: finite element analysis and biomechanical testing. Comput. Methods Programs Biomed. 234, 107502. 10.1016/j.cmpb.2023.107502 37003038

[B30] Nie S. Li J. Li M. Hao M. Wang K. Xiong Y. (2022). Finite-element analysis of a novel cephalomedullary nail for restricted sliding to reduce risk of implant failure in unstable intertrochanteric fractures. Orthop. Surg. 14 (11), 3009–3018. 10.1111/os.13497 36120825 PMC9627085

[B31] Nikoloski A. N. Osbrough A. L. Yates P. J. (2013). Should the tip-apex distance (TAD) rule be modified for the proximal femoral nail antirotation (PFNA)? A retrospective study. J. Orthop. Surg. Res. 8, 35. 10.1186/1749-799x-8-35 24135331 PMC3853127

[B32] Pfirrmann C. W. Metzdorf A. Zanetti M. Hodler J. Boos N. (2001). Magnetic resonance classification of lumbar intervertebral disc degeneration. Spine (Phila Pa 1976) 26 (17), 1873–1878. 10.1097/00007632-200109010-00011 11568697

[B33] Randelli F. Viganò M. Liccardi A. Mazzoleni M. G. Basile G. Menon A. (2023). Femoral neck fractures: key points to consider for fixation or replacement a narrative review of recent literature. Injury 54 (Suppl. 1), S70–s77. 10.1016/j.injury.2021.09.024 34615597

[B34] Ricci W. M. (2023). Stability of intertrochanteric femur fractures. J. Orthop. Trauma 37 (10s), S1–s4. 10.1097/bot.0000000000002675 37710368

[B35] Rinehart D. B. O'neill D. E. Liu J. W. Sanders D. T. (2021). Does size matter for cephalomedullary nails in geriatric intertrochanteric fractures? J. Orthop. Trauma 35 (6), 329–332. 10.1097/bot.0000000000001989 33079832

[B36] Rubio-Avila J. Madden K. Simunovic N. Bhandari M. (2013). Tip to apex distance in femoral intertrochanteric fractures: a systematic review. J. Orthop. Sci. 18 (4), 592–598. 10.1007/s00776-013-0402-5 23636573

[B37] Weil Y. A. Khoury A. Zuaiter I. Safran O. Liebergall M. Mosheiff R. (2012). Femoral neck shortening and varus collapse after navigated fixation of intracapsular femoral neck fractures. J. Orthop. Trauma 26 (1), 19–23. 10.1097/bot.0b013e318214f321 21904227

[B38] Weishaupt D. Zanetti M. Boos N. Hodler J. (1999). MR imaging and CT in osteoarthritis of the lumbar facet joints. Skelet. Radiol. 28 (4), 215–219. 10.1007/s002560050503 10384992

[B39] Xi Z. Xie Y. Chen S. Sun S. Zhang X. Yang J. (2023). The cranial vertebral body suffers a higher risk of adjacent vertebral fracture due to the poor biomechanical environment in patients with percutaneous vertebralplasty. Spine J. 23, 1764–1777. 10.1016/j.spinee.2023.08.003 37611873

[B40] Yang J. X. Luo L. Liu J. H. Wang N. Xi Z. P. Li J. C. (2024). Incomplete insertion of pedicle screws triggers a higher biomechanical risk of screw loosening: mechanical tests and corresponding numerical simulations. Front. Bioeng. Biotechnol. 11, 1282512. 10.3389/fbioe.2023.1282512 38260754 PMC10800439

